# Engineering the geometrical shape of mesenchymal stromal cells through defined cyclic stretch regimens

**DOI:** 10.1038/s41598-017-06794-9

**Published:** 2017-07-26

**Authors:** Brandan Walters, Tatiana Uynuk-Ool, Miriam Rothdiener, Julian Palm, Melanie L. Hart, Jan P. Stegemann, Bernd Rolauffs

**Affiliations:** 1 Department of Biomedical Engineering, University of Michigan, 1107 Carl A. Gerstacker Building, 2200 Bonisteel Blvd, Ann Arbor, MI 48109 United States; 2 Siegfried Weller Institute for Trauma Research, BG Trauma Clinic Tuebingen, University of Tuebingen, Waldhoernlestr. 22, 72072 Tuebingen, Germany; 3 G.E.R.N. Tissue Replacement, Regeneration & Neogenesis, Department of Orthopedics and Trauma Surgery, Medical Center - Albert-Ludwigs-University of Freiburg, Faculty of Medicine, Albert-Ludwigs-University of Freiburg, Hugstetter Straße 55, 79106 Freiburg, Germany; 4 Massachusetts Institute of Technology, Center for Biomedical Engineering, Cambridge, MA 02319 USA

## Abstract

Stem cells have been predicted to improve disease outcomes and patient lives. Steering stem cell fate - through controlling cell shape - may substantially accelerate progress towards this goal. As mesenchymal stromal cells (MSCs) are continuously exposed *in vivo* to a dynamically changing biomechanical environment, we hypothesized that exogenous forces can be applied for engineering a variety of significantly different MSC shapes. We applied specific cyclic stretch regimens to human MSCs and quantitatively measured the resulting cell shape, alignment, and expression of smooth muscle (SMC) differentiation markers, as those have been associated with elongated morphology. As proof of principle, a range of different shapes, alignments, and correlating SMC marker levels were generated by varying strain, length, and repetition of stretch. However, the major determinant of biomechanically engineering cellular shape was the repetition of a chosen stretch regimen, indicating that the engineered shape and associated differentiation were complex non-linear processes relying on sustained biomechanical stimulation. Thus, forces are key regulators of stem cell shape and the targeted engineering of specific MSC shapes through biomechanical forces represents a novel mechanobiology concept that could exploit naturally occurring *in vivo* forces for improving stem cell fate in clinical regenerative therapies.

## Introduction

Mesenchymal stromal cells (MSCs) are well known for their ability to differentiate *in vitro* into a wide range of somatic cells including osteogenic, chondrogenic, adipogenic, myogenic, endothelial, and neurogenic lineages^1–7^. MSCs are recognized as adult, self-renewing, and multipotent stem cells with substantial potential for therapeutic use^8, 9^. They have been forecasted to substantially change disease outcomes and patient lives^10^ and better understanding and controlling MSC properties could accelerate this goal substantially.

Cellular shape is a fundamental signal for proliferation^11^, potently regulates cell growth and physiology, and is indicative of specific functions^12^. Membrane protrusions influence cell shape and are highly relevant for adhesion, migration, and rigidity sensing^13^. Moreover, specific MSC shapes accompany the differentiation into different cell lineages, as rounded MSC shapes are associated with adipogenic differentiation and elongated shapes with myogenesis^14–17^. Utilizing this association of MSC shape with function, previous studies generated specific cell shapes for determining lineage commitment, using adhesive micro-patterned surfaces^18, 19^ and multi-perforated polycarbonate membranes^17^. Other studies have used cyclic tensile forces for inducing myogenic differentiation, while generating dynamically elongated cell shapes^16, 20^, based on the observation that elongated MSCs express markers of smooth muscle cells (SMCs)^17^. Thus, MSC shape will likely play an important role in understanding and engineering tissue constructs for future applications.

**Figure 1:**
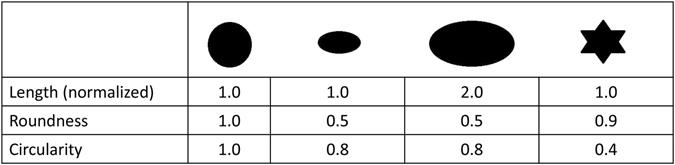
Comparison of Shape Factors Using Hyothetical MSCs. Figure 1 outlines the different features of cells that each shape factor defines. Cell length measures the “long axis” of each cell and has been used frequently in myogenic studies as cells undergoing differentiation become longer. Cell roundness is a ratio of “area” to “long axis” normalized to one. This quantitative measurement can be used to describe the rate of hypertrophy of cells with respect to both their short and long axes^55^. Cells with different roundness values could plausibly have similar lengths or vice versa. Circularity describes a third biologically relevant feature as it mathematically measures the ratio of “area” to “perimeter”, normalized to one. As cells spread or begin to migrate through their surroundings, they protrude out^13^. This may or may not be captured by changes cell length or roundness but, according to our definition, can drastically decrease circularity.

Previously, we demonstrated that the geometrical shape of many MSCs can be measured by quantitatively calculating mathematical shape descriptors with a semi-automated high-throughput method^21^. These shape descriptors describe different aspects of cell morphology (Fig. 1). Using this method and a system of competing cues for influencing MSC shape (with dynamic effects on shape through cyclic stretch and static effects on shape through the stiffness-defined biomaterial), we discovered that stretching cells did not necessarily produce elongated MSCs; instead, it produced MSCs that were ultimately rounder than unstretched controls^21^. In the present study we asked the fundamental question whether cyclic stretch regimens can be used for engineering a variety of defined cell morphologies, whether elongated MSCs can be generated with this approach, and what the impact on SMC marker expression would be. These questions are important, as stem cells are continuously exposed to a dynamically changing mechanical environment^22^, which acts as a key regulator of their fate^22, 23^, and because producing a variety of shapes through biomechanical forces could theoretically be utilized *in vivo* for controlling MSC function. Our general hypothesis was that varying parameters including maximum strain, stretch time, and the repetition of optimized stretch regimens (stretching the same specimen with the same parameters on two consecutive days) would generate significantly different MSC morphologies, and that varying these parameters could be used for specifically producing an elongated MSC shape. Consequently, we applied specific regimens of cyclic stretch to human bone marrow derived MSCs seeded on compressed collagen sheets (matched with nanoscale stiffness for myogenic differentiation^24^) and assessed the effects of this stimulus on cell phenotype. For assessing the impact of cell shape on phenotype, we investigated the expression of SMC markers as a function of stretch and respective morphology. Elongated MSC morphologies have typically been associated with increased expression of SMC markers^16, 17^, and because biomechanical forces increase MSC differentiation towards a SMC phenotype^14–16^, we expected these responses to correlate. Finally, because cyclic stretch is known to affect the alignment of cells relative to the stretch direction^14–16, 20, 25–27^, we asked how cyclic stretch affects MSC alignment and if these changes can be explained by cell morphology. Collectively, we aimed to introduce the novel concept of the targeted engineering of MSC shape through defined cyclic stretch regimens; this would advance our understanding of cell differentiation and promises broad *in vitro* and *in vivo* applications in mechanobiology, tissue engineering, and clinical regenerative medicine.

## Results

### Shape Descriptor: Cell Length

**Figure 2:**
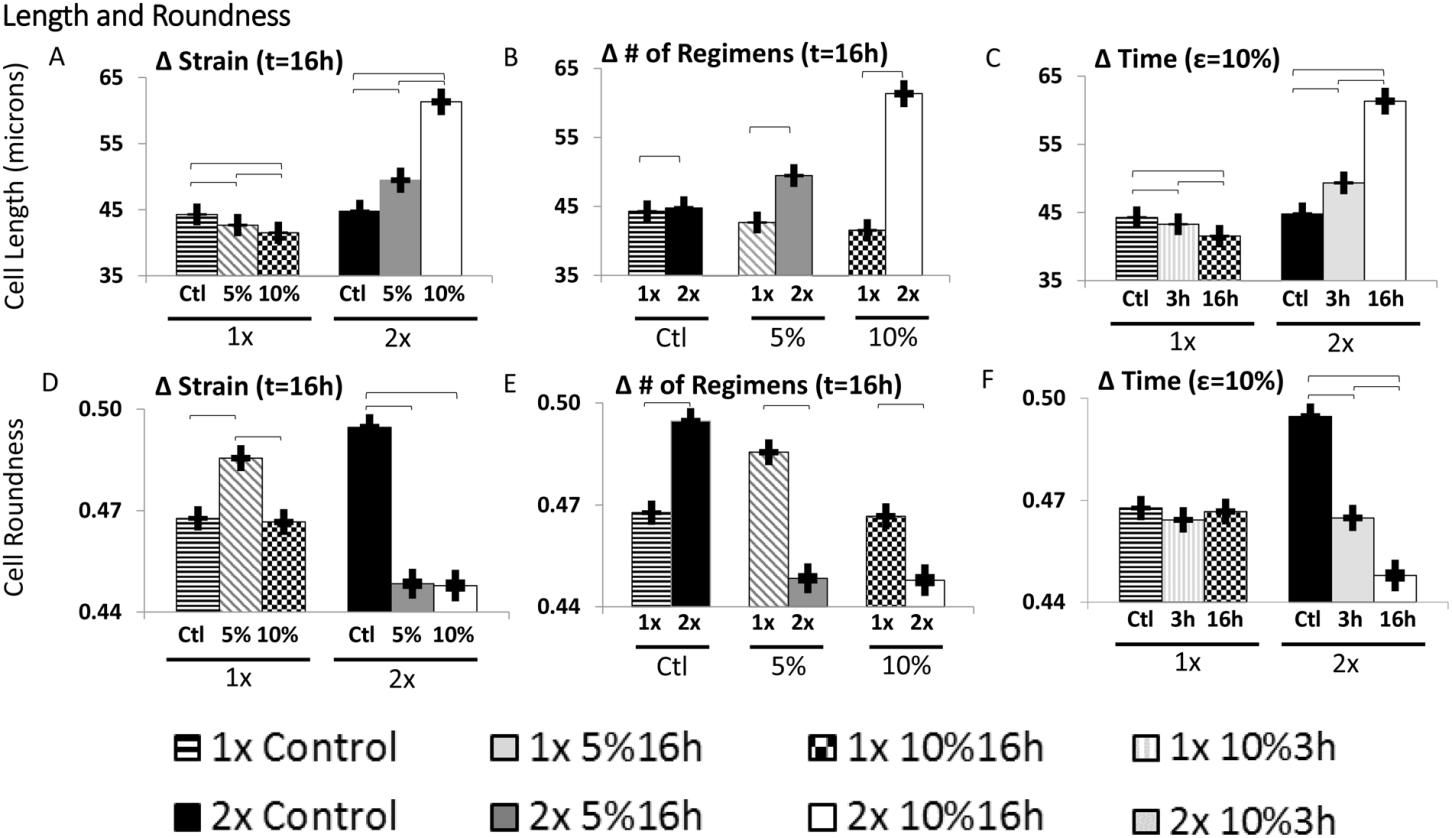
Effects of Stretch Parameters on Cell Length and Roundness. Stretching the cells at higher strains produces cells that are shorter after one regimen (p < 0.05) but longer after two (**A**), (p < 0.05). Regardless of whether or not cells are stretched, they are longer when cultured for an additional regimen (**B)**, (p < 0.05). After one stretch regimen, cells that are stretched longer periods of time are shorter (p < 0.05), however after a second regimnen of stretch, increased duration of stretch results in longer cells (**C)**, (p < 0.05). Stretching the cells for one regimen produces round cells at low strains (p < 0.05) but less round cells at high strain (**D)**, (p < 0.05). Cells that are stretched for two regimens are much less round regardless of strain (**D)**, (p < 0.05). If cells are left unstretched, they naturally round up but if they are stretched, additional regimens decrease roundness regardless of maximum strain (**E)**, (p < 0.05). Stetching cells for longer durations has no effect on cell roundess after only one regimen but after a second regimen, longer durations of stretch produce less round cells (**F)**, (p < 0.05).

Unstretched control MSCs increased their cell length over time but the change was small. After applying one regimen of cyclic stretch, cell length of stretched cells was shorter than that of unstretched control MSCs, regardless of the stretch parameters (p < 0.001). Increased strain during the first stretch regimen decreased MSC length (Fig. 2A: 1 × 5% vs. 10%; p < 0.05), and increased stretch time also decreased MSC length (Fig. 2C: 1 × 10% 3 h vs. 16 h; p < 0.05). These trends were completely reversed after applying a repeated second regimen of cyclic stretch the following day. A second regimen of stretch the next day generated a longer cell length than one regimen for all stretch parameters including controls (Fig. 2B: 1× vs. 2× Control, 1× vs. 2× stretch with 5%, 1× vs. 2× stretch with 10% 16 h; p < 0.05). This effect was amplified with higher strains (Fig. 2B; p < 0.05) and longer stretch time (Fig. 2C: compare 1× vs. 2 × 10% for 3 h to 1× vs. 2 × 10% for 16 h; p < 0.05). Stretching the cells for longer durations has similar effects as stretching at different strains. After one regimen, increasing the duration of strain decreased cell length (Fig. 2C: 1 × 10% 3 h vs. 16 h; p < 0.05) but after a second regimen, longer durations produced longer cells (Fig. 2C: 2 × 10% 3 h vs. 16 h; p < 0.05). The shortest MSCs were in populations that had been stretched at high strains for a single regimen and the highest MSC length was observed after two regimens of 10% stretch for 16 h. This was the highest strain applied for the longest period of time.

### Shape Descriptor: Cell Roundness

Next, we analyzed the effect of stretch on cell roundness. With a single stretch regimen, MSC roundness was either increased (Fig. 2D: 1× Control vs 5% for 16 h; p < 0.05) or was statistically the same as control populations (Fig. 2D: 1× Control vs 10% for 16 h). After the first stretch regimen, increased strain decreased MSC roundness (Fig. 2D: 1 × 5% vs. 10% for 16 h; p < 0.05) but increased stretch time had no effect on MSC roundness (Fig. 2F: 1 × 10% for 3 h vs. 16 h). Generally, control cells were rounder than cells stretched for a repeated regimen the next day (Fig. 2D; p < 0.05) but this decrease in roundness was not strain-dependent (Fig. 2D: 2 × 5% vs. 10% for 16 h). While unstretched MSCs increased in roundness over time (Fig. 1E: 1× vs. 2× Control; p < 0.05) stretched MSC roundness values were lower than after one stretch regimen (Fig. 2E: 1× vs. 2 × 5% for 16 h and 1× vs. 2 × 10% for 16 h; p < 0.05). Unlike with cell length, different responses were seen in roundness changes when using different strains and different durations. After one regimen, increasing the duration of stretch had no effect on cell roundness (Fig. 2F; 1× Control vs. 10% for 3 h vs. 10% for 16 h) but after a second, repeated regimen, increasing the duration decreased roundness (Fig. 2F: 2 × 10% for 3 h vs. 16 h; p < 0.05). Overall, the lowest MSC roundness was observed after two regimens of 5% and 10% stretch for 16 h, which was the longest period of stretch time. The cells with the highest roundness were unstretched controls statically cultured throughout the time needed for two stretch regimens.

### Shape Descriptor: Cell Circularity

**Figure 3:**
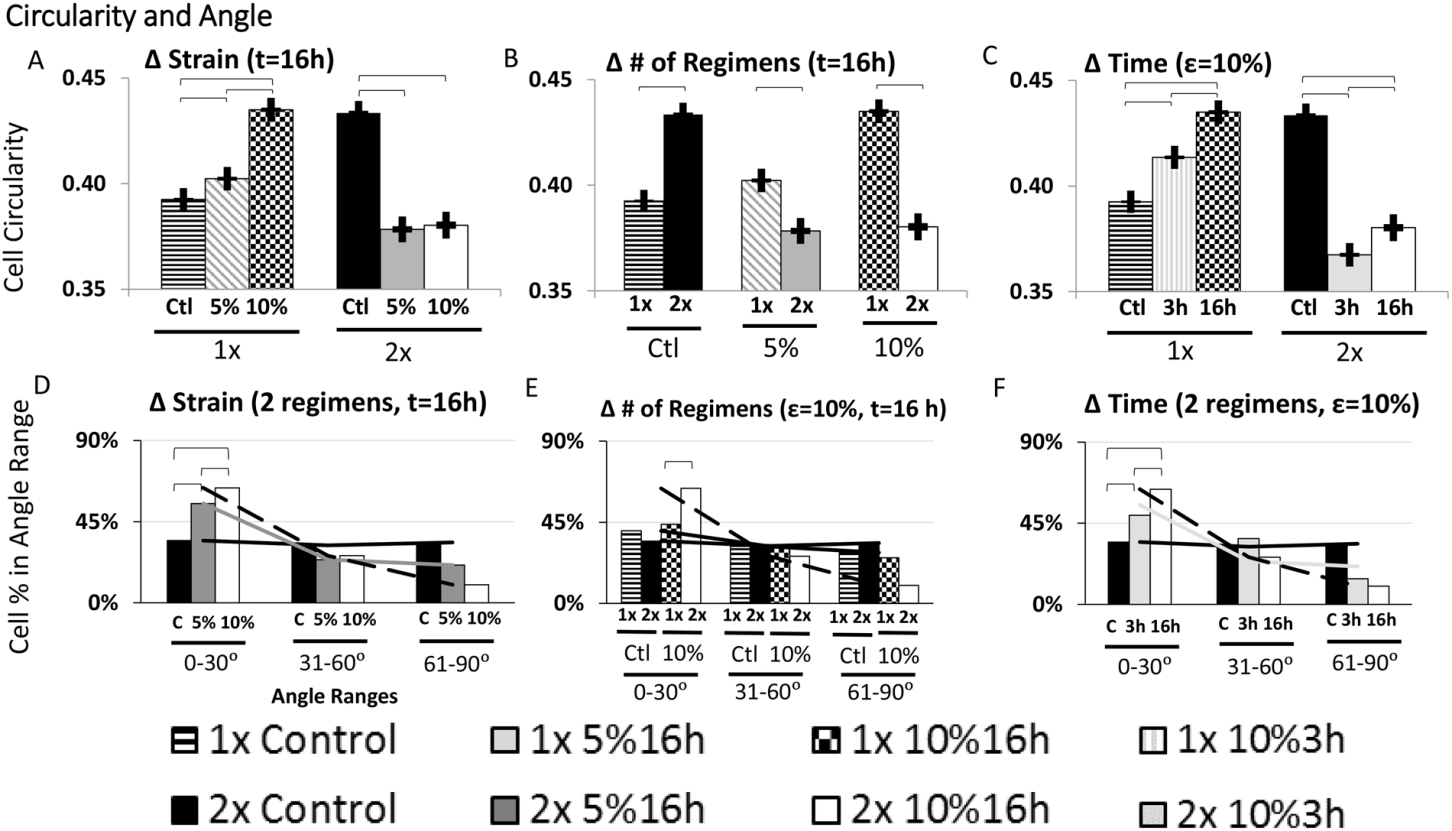
Effects of Stretch Parameters on Cell Circularity and Orientation. Cells stretched with 1 regimen have increased circularity and increasing the strain exagerates this effect (**A)**, (p < 0.05) but after a second regimen, cells that are stretched have lower circularity than controls (p < 0.05) but strain has no effect (**A**). Control cells have significantly higher circularities with additionnal regimens (p < 0.05) but cells that are stretched have lower circularity after a second stretch regimen (**B)**, (p < 0.05). After one regimen, cells stretched for longer durations have higher circularity (p < 0.05) but after a second regimen, they are much less circular than controls (**C)**, (p < 0.05). Increasing the duration of stretch increases circularity after either one or two regimens of stretch (**C**), (p < 0.05). Stretching cells at higher strains produces cells that more aligned (**D)**, (p < 0.05). Control cells are more aligned after additional culture time (p < 0.05) and repeating a stretch regimen produces cells that are much more aligned (**E)**, (p < 0.05). Increasing the length of time cells are stretched increases their alignment (**F)**, (p < 0.05).

We analyzed MSC circularity next. After a single stretch regimen, MSC circularity was higher than controls (Fig. 3A: 1× Control vs. 5% for 16 h; p < 0.05) and this difference increased with strain (Fig. 3A: 1 × 5% vs. 10% for 16 h; p < 0.05). After a second repeated stretch regimen the following day, stretched cells were less circular than controls but the amount of strain had no effect on cell circularity (Fig. 3A: 2× Control vs. 5% for 16 h and 2× Control vs. 10% for 16 h; p < 0.05). Unstretched control MSCs increased their circularity with time (Fig. 3B: 1× Control vs. 2× Control; p < 0.05) but stretching the cells inverted this response ubiquitously decreasing the cell circularity of cells stretched for two regimens compared one stretch regimen (Fig. 3B: 1× vs. 2× stretch with 5% for 16 h and 1× vs. 2× stretch with 10% for 16 h; p < 0.05). After one regimen, increasing the length of time for each regimen also increased the resulting circularity, even over respective controls (Fig. 3C: 1× Control vs 10% for 3 h and 1 × 10% 3 h vs 16 h, p < 0.05) before having overall lower circularity than controls after a second regimen (Fig. 3C: 2× Control vs 2 × 10% 3 h, 2× Control vs 2 × 10% 16 h, p < 0.05). Overall, the lowest circularities were observed in cells stretched for two regimens of stretch while the length of the stretch regimen and the strain had less significant impacts. The highest circularities were observed in cells left unstretched or cells stretched with high strain for a single regimen.

### MSC alignment relative to stretch

**Figure 4:**
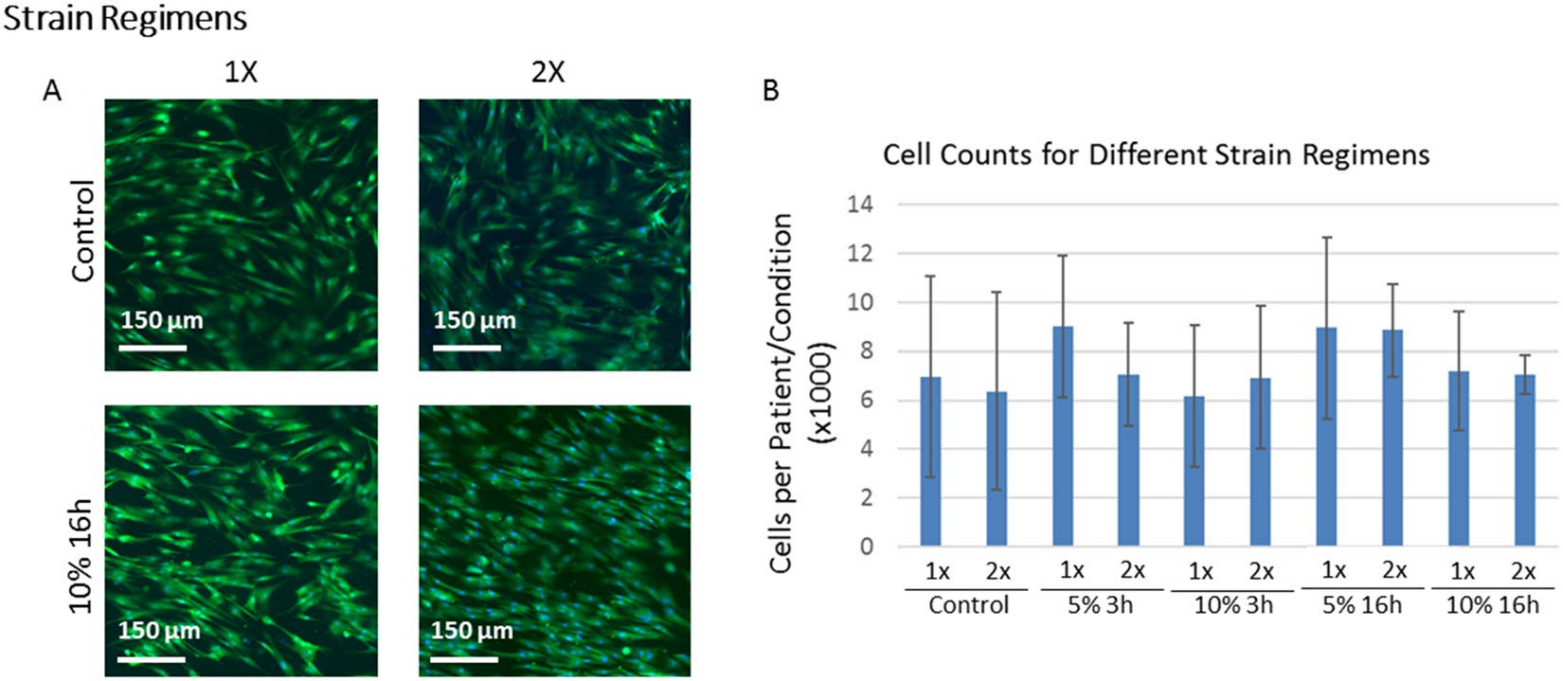
Shape and Cell Count of Stretched and Unstretched MSCs. Stretched and unstretched MSCs that adhered to the collagen type I sheets were stained with Calcein to illustrate MSC shape (**A**). Cell nuclei stained with DAPI (not shown) were counted to measure cell numbers (**B**). The axis of stretch was along the horizontal direction. Clear differences in MSC alignment can be seen in stretched vs. unstretched MSCs, and alignment was more pronounced in 2× than 1× stretched MSCs (**A**). More subtle differences in cell size and cell spreading were also present and quantified elsewhere. The number of MSCs across the experimental conditions tested was not statistically different. Error bars indicate standard deviation.

To assess the effects of cyclic stretch on cell alignment, we quantified the angle of the major cell axis relative to the direction of stretch or, in case of control MSCs, relative to the horizontal axis of the image. Stretching generally increased alignment parallel to the direction of stretch (Fig. 3D–F, p < 0.05). Stretching the MSCs at higher strains (Fig. 3D: 2 × 5% vs. 10% for 16 h; p < 0.05) or for longer durations (Fig. 3F: 2 × 10% for 3 h vs. 16 h; p < 0.05) resulted in more alignment from MSCs. Even cells stretched the highest strain and longest duration had statistically similar angles as controls after a single regimen (Fig. 3E: 1× Control vs. 1 × 10% for 16 h). Regardless of the other parameters, it required a repeated stretch regimen on the next day to induce alignment and unstretched control MSCs remained isotropic regardless of culture time (Fig. 3E). Thus, stretch consistently induced MSC alignment and the degree of alignment corresponded to the amount of stretch but interestingly, this effect was only visible after two regimens of stretch on day 4 and 5. The stretch-induced alignment is illustrated in Fig. 4A.

### MSC Numbers

Stretched and unstretched control MSCs were stained and counted. There were no statistical differences in the number of MSCs analyzed (Fig. 4B).

### MSC mRNA Expression

**Figure 5:**
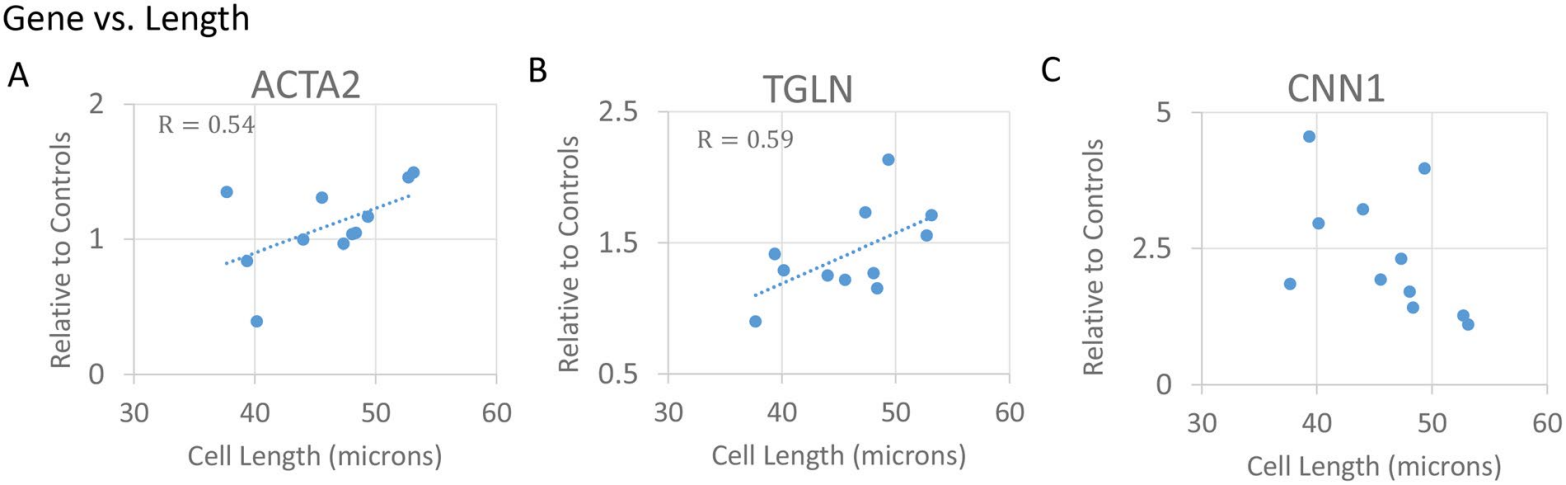
Correlation of Cell Length with Smooth Muscle Gene Expression. Cell length positively correlates significantly with ACTA2 mRNA expression of the population independently of stretch regimen (**A)**, (R = 0.54, p < 0.05). Cell length significantly and positively correlates with TGLN expression of the population regardless of stretch parameters (**B)**, (R = 0.59, p < 0.05). There is no significant correlation between cell length and CNN1 gene expression (**C**).

As shape descriptors have been shown previously to correlate with the expression of specific smooth muscle markers^21^, we asked if cell length had similar correlation and if these relationships were impacted by the stretch regimen. Using q-PCR and applying the above reported stretch regimens, we demonstrated that MSC length correlated with smooth muscle ACTA2 regardless of stretch regimen (Fig. 5A: R = 0.54; p < 0.05) as did TGLN mRNA expression (Fig. 5B: R = 0.59; p < 0.05), which are respectively early and intermediate gene markers for smooth muscle differentiation^28^. Interestingly, MSC length did not correlate with the expression of CNN1, another intermediate marker for smooth muscle differentiation (Fig. 5C).

### MSC Protein Expression

**Figure 6:**
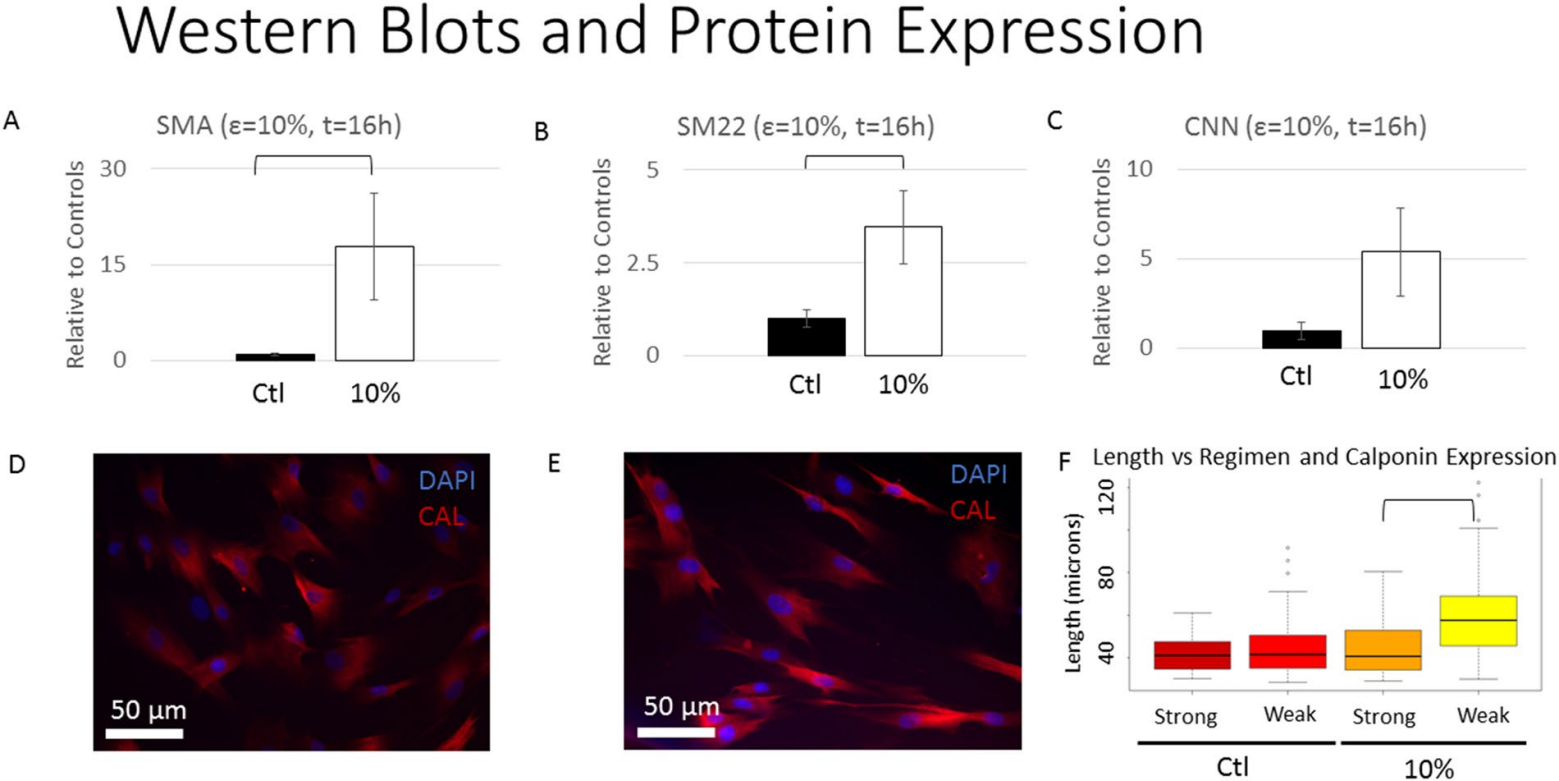
Effects of Stretch on Smooth Muscle Protein Expression. Western blots reveal that cells stretched to 10% for two 16 hour regimens expressed more SMA protein than controls (**A)**, (p < 0.05). Cells stretched to 10% for two 16 hour regimens also expressed more SM22 protein (**B)**, (p < 0.05). There was no statistical difference in CNN protein expression between cells stretched to 10% and their respective controls (**C**). Cells stretched at the maximum strain (10%) and duration (2× for 16 hours) as well as their respective control were stained with anti-calponin ((**D)**: controls; (**E)**: stretched MSCs) and the length of cells with a bright signal (above a specific pixel intensity threshold) was compared to that of cells with a weaker signal (**F**). Visualizing the cell contours up close, the majority of cells with a strong Calponin signal appeared shorter than longer appearing MSCs with a weaker Calponin signal. There was no statistical difference between the length of control cells expressing strong CNN signal or cells expressing weak amounts of protein. Stretched cells that exhibited weak expression of CNN were significantly longer than their strongly expressing counterparts (p < 0.05).

While the expression of ACTA2 and TAGLN correlated well with MSC length, we wanted to further examine if expression of CNN1 protein related to MSC length. We chose the stretch regimen that generated the highest MSC length (2 × 10% for 16 h) and compared the induced protein expression with their respective unstretched MSC controls, asking whether MSCs, whose length was generated through cyclic stretch, would generate more protein. Western Blots revealed that MSCs stretched with 2 × 10% for 16 h expressed more SMA, the protein transcribed from ACTA2 (Fig. 6A, p < 0.05), and SM22, the protein transcribed from TAGLN (Fig. 6B, p < 0.05), than their respective controls. CNN protein expression did not exhibit a statistically significant relationship (Fig. 6C).

Because CNN1 mRNA expression and cell length did not correlate, and the CNN protein in western blots did not exhibit a statistically significant relationship to stretch, we investigated the expression of the protein through fluorescence microscopy. We noted that the fluorescence of CNN antibodies of MSCs stretched 2 × 10% for 16 h was not homogeneous. We asked whether CNN protein expression could be related to MSC length on an individual cell basis. We fluorescently stained the cells for CNN and investigated how the length of MSCs with a higher intensity (selected based on signals above a chosen threshold) differed from the length of MSCs with a lower CNN fluorescent intensity (Fig. 6D and E). Stretched MSCs did have a higher proportion of MSCs with “high” CNN expression compared to unstretched controls (36.9% vs 17.6%). However, we demonstrated that stretched MSCs expressing “high” CNN were statistically shorter (Fig. 6F,D, p < 0.05) and had similar lengths to controls. In unstretched populations, cells with higher expression and lower expression had statistically similar lengths. Thus, while both the qRT-PCR and immunohistochemistry data suggested that stretch influenced the expression of CNN, increased expression seems to be unassociated with cells lengthening.

### Associations of MSC shape and alignment

**Figure 7:**
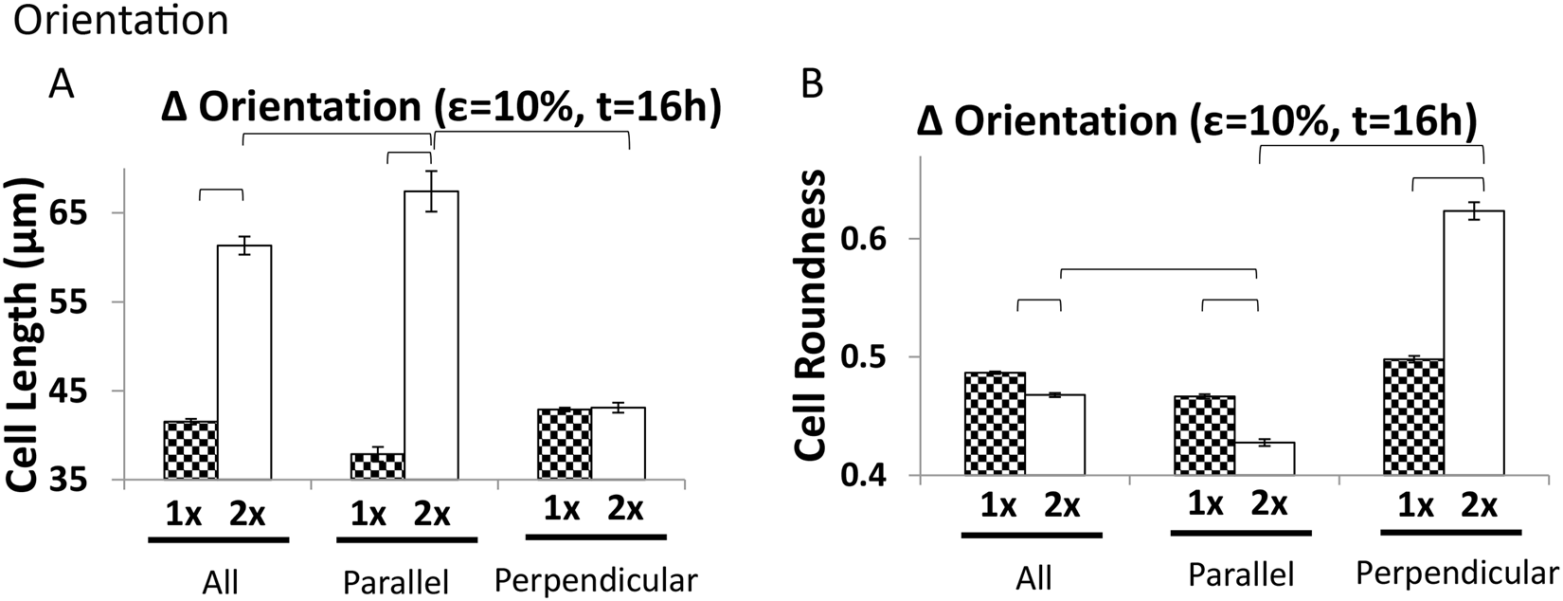
Influence of Orientation on Stretch Induced Changes in Morphology. When cells are stretched for a second regimen, the average cell length of the entire population would increase (**A)**, (p < 0.05). By looking specifically at cells oriented in parallel with the direction of stretch, their change in length between the first and second cycle is even greater (**A)**, (p < 0.05). When considering the cells oriented perpendicular to the direction of stretch, a second regimen does nothing to the cell length (**A**). Looking at cell roundness shows that a second regimen of 10% stretch produces cells that are less round than those only stretched for a single regimen (**B)**, (p < 0.05). An exaggerated trend is seen in the cells solely oriented in parallel with the direction of stretch (**B**), (p < 0.05). When considering the cells oriented perpendicularly, the cells are rounder after a second regimen of stretch (**B)**, (p < 0.05).

Based on these findings, we asked whether MSC shape might be more dependent on alignment than stretch regimen. To investigate this, we examined the shape of cells aligned in specific directions after any given treatment. We compared MSCs with an alignment parallel to the direction of stretch (0–30°) vs. MSCs perpendicular to the direction of stretch (61–90°). As we expected from our assessment of cell angle, not only were cells isotropic after a single round of stretch, the shapes of cells were similar regardless of orientation (Fig. 7A and B). After a second regimen of stretch the next day however, differences arose and interesting patterns resulted. We saw that cells that aligned parallel to the direction of stretch became much longer than cells in same orientation after one regimen of stretch (Fig. 7A: Parallel 1 × 10% 16 h vs. 2 × 10% 16 h, p < 0.05), and that these parallel aligned MSCs were longer than the average cell length of the entire population (Fig. 7A: Parallel vs. All 2 × 10% 16 h, p < 0.05). The perpendicular cells were no longer after a second regimen of stretch than they were after a single stretch regimen (Fig. 7A: Perpendicular 1× vs. 2 × 10% for 16 h). In contrast, after two stretch regimens they were shorter than the parallel and total cell population lengths (Fig. 7A: All vs. Perpendicular 2 × 10% for 16 h and Parallel vs. Perpendicular 2 × 10% for 16 h; p < 0.05). Thus, the change in MSC cell length in relation to stretch depended on initial MSC orientation. At the same time, after two regimens of stretch repeated on consecutive days, parallel aligned MSCs were less round compared to the entire population (Fig. 7B; p < 0.05) while the perpendicularly aligned MSCs were much rounder than the parallel MSCs (Fig. 7B: Perpendicular 2 × 10% 16 h vs. Parallel 2 × 10% 16 h; p < 0.05) and also much rounder than the entire population (Fig. 7B Perpendicular 2 × 10% 16 h vs. All 2 × 10% 16 h; p < 0.05). Overall, this suggested that two regimens of stretch were necessary to induce changes in alignment and that the cell shapes changed throughout this alignment process. For parallel cells, an additional regimen increased cell length and decreased roundness while perpendicular cells became rounder as they elongated along the direction of stretch. Collectively, this suggested that MSC morphology induced by cyclic stretch depended on cell orientation, and, in turn, that alignment played an instrumental function in generating MSC morphology through cyclic stretch.

## Discussion

The application of cyclic stretch has actively been used for inducing MSC differentiation into specific lineages^16, 24, 29–32^, and also for generating an elongated cell morphology^29^. While this has made cyclic stretch an important and widely used tool in tissue engineering, we recently discovered that stretching a population of randomly aligned, initially isotropic, MSCs generated a rounder phenotype and that this roundness was the result of a complex shape remodeling process that occurred during stretch and that continued afterward^21^. Here, we hypothesized that variations of parameters such as strain, stretch time and the repetition of stretch regimens would generate significantly different MSC morphologies including elongated MSCs, as this is commonly one of the major goals for applying cyclic stretch. Although variations of strain and time did indeed produce different morphologies, they were not the major determining factor. Instead, a second regimen of stretch repeated the next day was associated with more alignment and an elongated phenotype indicative of myogenic differentiation. Our results suggest this second repetition is the minimum number of required regimens to induce alignment with the direction of stretch and initiate the morphological phenotype associated with differentiation and smooth muscle maturation. Thus, variations of strain and length of stretch regimen were modulators, which intensified or attenuated the effects of the respective stretch regimen. Future studies could investigate the effect of additional stretch regimens by applying more than two consecutive runs. Collectively, these findings confirmed our general hypothesis that regimens of cyclic stretch can be used for engineering specific MSC morphologies with distinct aspects of shape quantified by length, roundness, and circularity.

With the goal to produce a controlled range of contrasting MSC morphologies, we asked which experimental conditions, including control culture conditions, would affect cell shape and orientation. The highest absolute length and lowest roundness values represented a long, thin MSC that has been typically associated with a myogenic phenotype^16, 17^. We confirmed this association and demonstrated that we can engineer a long, thin MSC morphology with two regimens of 10% stretch for 16 h repeated on consecutive days. We also found that these conditions led to low circularity that indicated cell spreading. In complete contrast to long, thin MSCs, we also engineered a short, round morphology that fits with the traditional views of undifferentiated cells. Those were generated with applying stretch only briefly, as the smallest MSC length was generated with 1 regimen of 10% stretch for 16 h, and by not applying stretch, as highest roundness and lowest aspect ratio occurred in non-stretched controls over time. These data also highlighted the relationship between biomechanically engineered shape, biomaterial choice, and select biochemical phenotypes. For this study, we used stiffness-optimized compressed collagen type I sheets with a concentration of 80 mg/ml because these sheets were associated with the most SMC-like phenotype among a range of (unstretched) biomaterials^21^. However, we confirmed that tuned stretch was even more effective in producing a long, thin MSC morphology than the compressed collagen sheets alone. This is relevant for engineering environments to biomechanically induce specific lineages of differentiation for MSCs. Next, we specifically demonstrated that the mRNA expression of ACTA2 and TAGLN correlated with the biomechanically generated cell length, and that the expression of analogous proteins, SMA and SM22, were significantly higher after the protocols producing the longest MSC morphology. While one interpretation of this data is that the upregulation of ACTA2 and SMA is a sign of cell shape change and alignment rather than maturation an indication of differentiation itself (ACTA2 and SMA have been associated with cell cytoskeleton remodeling^33, 34^), the continuous elongation and decrease in roundness and circularity suggest increased expression beyond stages of cell reorientation. These data imply that additional stretch and a more elongated MSC would promote the upregulation of ACTA2 and TAGLN along with continued expression of SMA and SM22, leading to a more mature phenotype. Interestingly, CNN1 mRNA expression did not correlate with cell length and CNN protein expression was not statistically upregulated in stretched cells. In fact, we observed that shorter cells in the stretched population expressed more CNN than longer cells. This would imply that not all SMC markers necessarily correlate with cell length and that other morphological features, like roundness, may be better indicators of differentiation, as their relationship to stretch parameters (Fig. 2) and genes^21^ is clearer. We have focused our investigations on smooth muscle differentiation because elongated MSC morphologies are firmly associated with an increased expression of SMC markers^16, 17^, and because biomechanical forces increase MSC differentiation towards a SMC phenotype^14–16^. However, the applications of engineering cell shape through biomechanical tensile forces extend beyond myogenic studies. Cell length is one of the few shape descriptors that has been actively quantified and associated with differentiation^17, 35, 36^ and function of SMCs^37^ but the control over additional shape factors introduced here are applicable in countless other studies pertaining to cell function and/or lineages of differentiation. Whether shape can potentially be engineered without simultaneously modulating MSC differentiation is an interesting question and remains to be answered. We are currently designing a system to isolate these stimuli and determine if stretch and growth factor cocktails can induce differentiation despite restricting and controlling a cell’s shape.

While this work has contributed to discerning the role of various stretch parameters on the change in MSC morphology and phenotype, it also raises additional questions that need to be addressed in future studies. We kept the final timeline of regimens with different strains and durations consistent, which led to differences in relaxation time. Having constant endpoints meant we could not account for these differences in relaxation time. As the collagen substrates and cells themselves are viscoelastic in nature and relaxation times has effects on cell morphology^38^, altering the relaxation time between regimens could prove interesting. This could be investigated by keeping constant stretch and relaxation times, which would result in differences in experimental length. However, in this investigation we were more concerned with comparing the effects of strain and duration of stretch and, thus, decided to keep the final endpoints consistent. We also kept the frequency during the stretch cycles consistent at 1 Hz, based on the literature, as previous studies applying cyclic stretch to MSCs have used a frequency of 1 Hz^14, 20, 39^. However, one study compared different frequencies and demonstrated that 1 Hz vs. 2.75 Hz had no significant effect on MSC alignment but on the cell shape index, which is comparable to the here used roundness. Interestingly, 2.75 Hz at 1% strain exhibited the same trend as a frequency of 1 Hz at 5% and 10% strain^16^, as applied here. Nevertheless, it could very reasonably be conjectured that the strain rate impacts the phenotype of stretched cells as this is directly linked to the material and cell viscoelastic properties. We may have seen this phenomenon ourselves; to achieve 10% strain in the same amount of time as 5% strain, the average strain rate is effectively doubled. It could very well be that this difference drives the here reported responses to stretch and not the maximum strain itself. Future investigations could investigate this effect by decoupling maximum strain from strain rate. However, matching sinusoidal wave functions for change in rates could prove challenging. Answering this question may require adapting a sawtooth function for comparison across different frequency spaces. Another question that arises out of this work is how individual cells are affected by stretch. We have developed means to examine how the shape of individual cells is changed in response to biomechanical stimuli but our biochemical analyses of these cells has been on the population as a whole. It would be quite useful to examine how individual cells are responding to heterogeneity in binding to a non-homogenous material and how these disparities manifest in differences in the expression of differentiation markers on mRNA and protein levels. E. g. single cell PCR or even studying the development of single cell focal adhesions could help determine influences of heterogeneous strain on MSCs.

**Figure 8:**
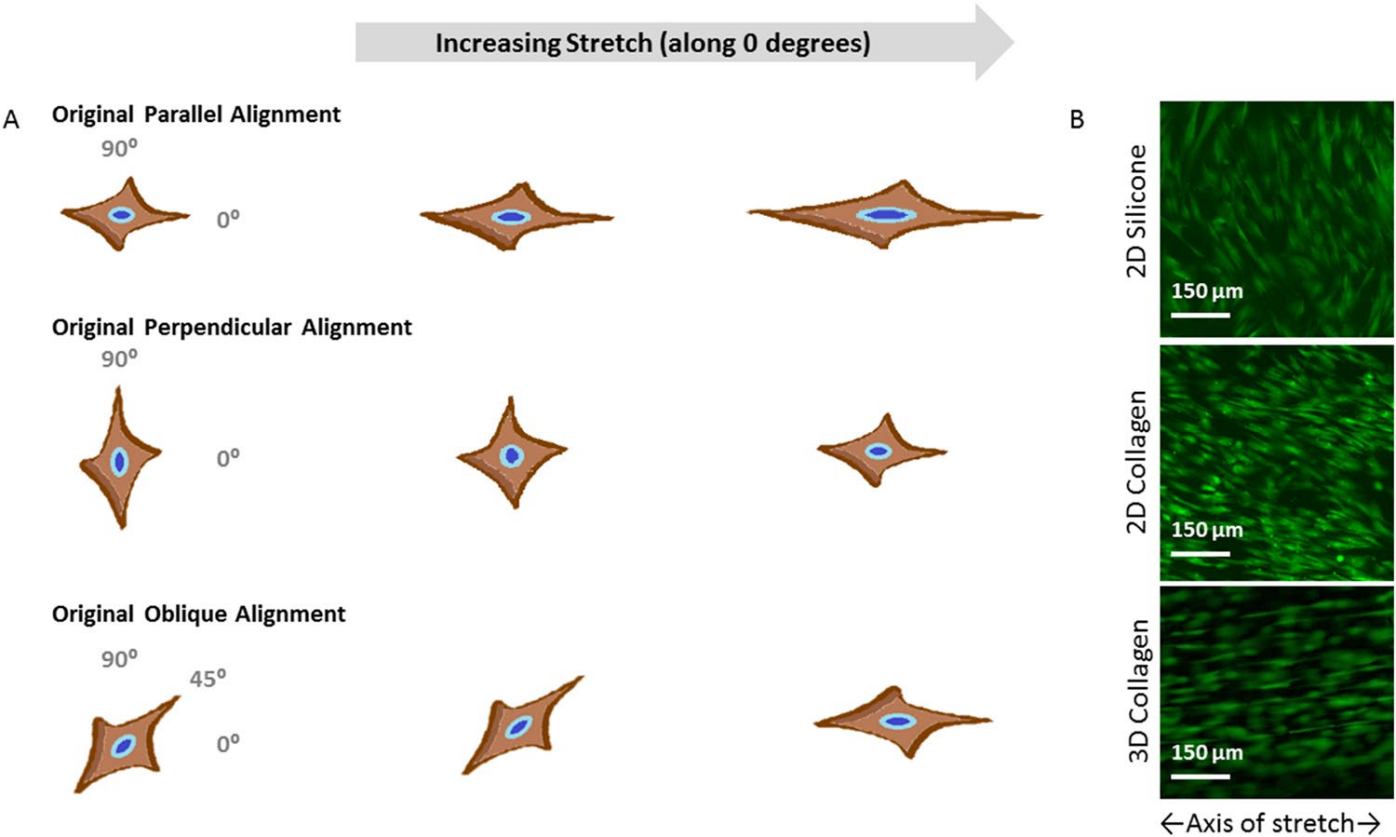
Change of MSC Shape as a Function of Cell Orientation, Stretch Direction and Substrate. Here are three schematic representations of the changes that 3 hypothetical MSCs undergo as a result of stretch and initial orientation (**A**). Cell nuclei are in blue and cell outlines are in brown color and star-like to visualize the exaggerated changes in shape parallel and perpendicular to the direction of the applied stretch, which is left-to-right, horizontally. With parallel cell alignment (see the first row with the left most cell pointing to 0 degrees), an MSC becomes longer in the direction of stretch while simultaneously contracting perpendicular cell extensions. This change increases the length and decreases both circularity and roundness. With perpendicular cell alignment (see the second row with the left most cell pointing to 90 degrees), a single MSC first contracts in the perpendicular direction and extends along the direction of stretch, eventually looking like it simply reoriented. This change produces a rounder and more circular cell with a decreased length before additional stretch returns its shape to an elongated morphology. With an oblique cell alignment (see the third row with the left most cell pointing to 45 degrees), the elongated MSC is stretched and it contracts protrusions away from 90 degrees and elongates in the direction of stretch. Mathematically, this follows the perpendicular cell but to an exaggerated extent. Collectively, the morphological changes of these 3 hypothetical MSCs highlight how stretching differently aligned MSCs can change their resulting morphology. The next panels exhibit the differences in alignment and shape induced by seeding on different substrates (**B**). MSC seeded on collagen coated silicone aligned perpendicularly to the direction of stretch while MSC seeded at the same concentration on a collagen sheet aligned parallel to the direction of stretch. When the cells were embedded in a 3D hydrogel of collagen and stretched, the cells aligned parallel to the direction of stretch.

Another important finding of the present study was the effect of cyclic stretch on MSC alignment and corresponding shape. Alignment has been shown to be important for MSC differentiation^40^ but when dissecting the pronounced effect of two consecutive stretch regimens into effects on parallel vs. perpendicularly aligned MSCs, it became obvious that parallel aligned MSCs drastically increased in cell length but perpendicular aligned cells did not. To better understand these unexpected results, it is necessary to describe this stretch response in more detail as a function of the initial cell alignment prior to stretch. Cells that were coincidentally aligned parallel to the stretch direction elongated along the axis of stretch and contracted any protrusions perpendicular to the direction of stretch. Quantitatively, this resulted in cells being longer, less round, and less circular with additional stretch. The cells that coincidentally started oriented perpendicular to stretch contracted their perpendicular axis and started to elongate along the axis of stretch, resulting in a seemingly reoriented cell. Quantitatively, the first stretch regimen made the perpendicular MSCs shorter, rounder, and more circular, and this shape was similar to that of unstretched counterparts. Only a second stretch regimen increased the contraction along the perpendicular axis and the elongation along the axis of stretch, which made the MSCs quantitatively longer and less circular and round, explaining how alignment can have differential effects on biomechanically engineered shape. Importantly, the majority of MSCs were initially neither parallel nor perpendicular to the stretch axis. Because those cells initially contracted away from the perpendicular direction before elongating and reorienting along the axis of stretch, and because they made up the majority of the population, their behavior drove the average population values presented in our first results. Moreover, these data highlight that MSC alignment can theoretically be explained by changes in overall MSC morphology but not by length alone. Indeed, we found that isotropically plated MSCs, when stretched and analyzed as a single population, would pass through a phase where their morphology was rounded, more circular and shorter. Typically, this phase occurred after a single regimen of stretch and only after a second regimen of stretch would the MSCs begin to align with stretch and display the biomechanically engineered morphologies that are conventionally associated with the myogenic phenotype. To better visualize these complex changes in MSC morphology, it is helpful to consider 3 hypothetical MSCs with parallel, perpendicular and an oblique alignment (Fig. 8A). Because these data demonstrated collectively that engineering MSC shape through applying cyclic stretch depends highly on MSC alignment, and, in turn, that repeating the stretch regimen was necessary to control this alignment, one could consequently envision controlling cell alignment through biomaterial designs or other engineering controls to maximize the effects of stretch on MSC morphology and differentiation.

In this view, our findings extend considerably to the importance of using the appropriate biomaterial for stretching and engineering the shape of adherent cells. Many previous studies have reported that MSCs and SMCs align under uniaxial stretch. Within 3D fibrin hydrogels, cells have been shown to align parallel to stretch^15^ but on 2D protein-coated silicone, MSCs and vascular cells are reported to align perpendicular to the stretch axis^14, 16, 20, 25, 26^. It could be speculated that MSCs respond differently to forces when cultured in dissimilar dimensional conditions. We show here that when seeded on collagen, we can stimulate parallel MSC alignment on 2D substrates, a phenomenon previously seen with stem cells only in 3D. That being said, studies stretching osteosarcoma cells on 2D collagen substrates have also shown parallel alignment^27^. We briefly investigated this phenomenon and found corroborating results. When we plated MSCs on the surface of collagen type I coated silicone and stretched, they aligned perpendicularly to the axis of stretch (Fig. 8B) as in^14, 16, 20, 25, 26^. When seeded on the surface of 2D collagen sheets and stretched, MSCs aligned parallel to the axis of stretch (Fig. 8B), comparable to^27^. However, MSCs seeded within a 3D collagen hydrogel aligned parallel to the direction of stretch (Fig. 8B), as they did in fibrin gels^15^. Based on these results it is clear that the substrate places a pivotal role in the resulting cellular alignment. In^15^, the hypothesis is stated that this difference is stiffness-driven and suggests that tests with the same material and different stiffnesses would be needed for confirmation. Here, we suggest an alternative explanation: that it is the *type* of substrate which determines alignment. It is plausible within our reported results and those of others, that non-fibrous substrates like silicone evoke perpendicular alignment while fibrous substrates are conducive if not inductive to parallel alignment in response to mechanical stress. We base this theory on the results seen in Fig. 8, along with the similar mechanical properties that the here used collagen sheets have^21^, compared to thin silicone sheets. More experiments are needed to discern the exact cause, but regardless of the outcome, it is apparent by the data presented here that the substrate choice plays an instrumental role in the biomechanically achievable shape and differentiation potential of MSCs.

Another interesting extension to this work would be to study the development of focal adhesions and how cell binding influences shape, integrin signaling cascades, and differentiation. When examining the effects of ligand-cell affinity on smooth muscle differentiation, control of the surface area and cell aspect ratio were found to strongly influence this process^41^. Other groups have manipulated cell area^19^ and cell spreading^18^ to control subsequent stem cell differentiation by changing the area available for focal adhesions. Previously, focal adhesion development has also been linked to substrate stiffness^42^ and can also be stimulated by mechanical forces^43, 44^. These focal adhesions at integrin binding sites regulate the actin cytoskeleton and impact ERK/MAPK pathway^45^, which has been shown to be instrumental in myogenic maturation and stem cell differentiation^46–48^. Additional ways that ECM interactions influence stem cell fate are reviewed in ref. 49.

Finally, we have demonstrated in the present study that specific aspects of MSC shape change under cyclic stretch and can be captured by different shape descriptors. While a general emphasis on using cell length provides substantial insight into a changing phenotype^17, 35–37^, focusing solely on this morphological feature may neglect multiple other shape aspects. Therefore, we advocate using a panel of shape descriptors such as those used in this study or introduced in our previous study^21^ for quantitatively describing MSC shape.

In conclusion, engineering the geometrical shape of MSCs through applying cyclic stretch is possible and its strong impact on differentiation makes it a young and promising field. Variations of strain and duration of stretch regimen were not the major determinants of biomechanically engineering cellular shape; instead it was the repetition of the chosen stretch regimen, indicating that the engineered shape and differentiation are complex non-linear processes that rely on sustained biomechanical stimulation. Additionally, substrate choice and initial cell alignment played roles in the engineering of cell shape through biomechanical forces. We envision enhanced effects with the targeted design of clinically applicable force-transducing and, thus, alignment-supporting and shape-instructive biomaterials, which could even be conditioned prior to implantation using a brief period of stretch, as in this study. Moving forward, as stem cells are continuously exposed *in vivo* to a dynamic biomechanical environment, this here introduced concept will help us to fine tune the biomechanical engineering of cell shape and the associated phenotype, ultimately promoting better *in vivo* control of cells for use in regenerative therapies, by harnessing the naturally occurring *in vivo* forces in the body of the patient.

## Methods

### MSC isolation and expansion

Bone Marrow aspirates were obtained from the proximal femurs of osteoarthritic patients (n = 29, age 50 to 86) who underwent total hip replacement in the Department of Trauma and Restorative Surgery, BG Trauma Clinic, University of Tuebingen. Aspirates were taken with approval of the local research ethics committee of the Medical Faculty of the University of Tuebingen (623/2013BO2) and with informed consent from the patients. All methods were carried out in accordance with the guidelines of the local research ethics committee of the medical faculty of the University of Tuebingen. Human bone marrow MSCs were isolated as described in ref. 24. Aspirates were washed with PBS, centrifuged at 150 g (10 minutes, room temperature), and the pellet was re-suspended in PBS, discarding the supernatant. The MSC were then separated using a Ficoll density gradient fractionation (density 1.077 g/mL, GE Healthcare Life Sciences, Uppsala, Sweden, 400 g, 30 min, room temp). The mononuclear cell layer was removed, washed in PBS and seeded in T75 flasks. The separated MSC were cultured and expanded at 37 °C and 5% CO_2_ in good manufacturing practice (GMP)- compliant media made with DMEM low glucose (Sigma-Aldrich, Hamburg, Germany), 1000 IU heparin (Carl Roth, Karlsruhe, Germany), 25 mM hepes (Lonza, Basel, Switzerland), 5% human plasma (TCS Biosciences, Buckingham, UK), 5% human pooled platelet lysate (10^8^ platelets/mL medium, ZKT Tuebingen, Germany), 2 mM L-glutamine (Lonza), and 1% penicillin-streptomycin solution (Life technologies, Darmstadt, Germany), according to refs 7 and 50. 24 h after seeding, GMP expansion media was replaced and then changed twice a week. At near confluence, after around 7 days, cells were removed with trypsin, counted, and re-plated (10^4^ cells per flask) in GMP expansion media for further passaging.

### Expression of cell surface antigens

The expression of CD90, CD14, CD11b (R&D Systems, Minneapolis, USA), CD105, CD73, CD45 and CD34 (BD Pharmingen, San Diego, USA) on MSC was analyzed by flow cytometry as we have shown previously^6, 51^, and in accordance with^52^. MSCs were detached gently using Accutase. Unspecific binding of antibodies was blocked with Gamunex (Talecris Biotherapeutics, Frankfurt, Germany). The MSC were washed twice with PFEA buffer (PBS, 2% FCS, 2 mM EDTA, 0.01% sodium azide) and incubated for 20 min at 4 °C with phycoerythrin (PE)-conjugated or allophycocyanin (APC)-conjugated monoclonal antibodies (mAB, BD Pharmingen, Heidelberg, Germany). Unbound antibodies were washed away twice with PFEA buffer, and MSCs were analyzed by flow cytometry (BD LSRII, San Diego, USA). Data were evaluated using the software FlowJo (Tree Star, Inc., Ashland, Oregon, USA).

### MSC seeding on compressed collagen sheets

Compressed rat collagen I sheets with a previously optimized collagen concentration of 80 mg/mL^21, 24^ were generated (Amedrix, Esslingen, Germany) and cut into 4 × 1 cm sheets. After passaging in GMP expansion media (DMEM with pooled plasma and platelet lysate as described^21, 24^), MSCs (passages 3–5) were seeded at 15,000 MSC/cm^2^ at day 0 onto collagen sheets. Sheets were cultured in high glucose DMEM (4 g/L, Life Technologies, Darmstadt, Germany), 10% FBS (Biochrom), 2% penicillin-streptomycin (Gibco/Life Technologies), and 1% fungicide (Biochrom) at 37 °C and 5% CO_2_ for 3 days before stretching on day 4.

### Sinusoidal cyclic stretch of collagen sheets and adhering MSCs

Collagen sheets seeded with MSCs at passages 3–5 with 15,000 cells/cm^2^ were cultured for 3 days and inserted on day 4 into the bioreactor chamber of an incubator-housed ElectroForce 5210 BioDynamic-Test-System (Bose, Minnesota, USA). Identical bioreactor chambers filled with 200 mL of control media were used for applying cyclic stretch and for incubating unstretched control sheets. Uniaxial displacement-controlled cyclic stretch was applied at 1 Hz with either 5% or 10% strain and for either 3 or 16 hours on day 4 only, or on days 4 and 5 before analyses. Sheets stretched for a single regimen and their unstretched control sheets cultured for the same period of time were labeled “1×”. One half of this set of sheets was analyzed, the other half of this set of sheets was incubated overnight and stretched again on day 5 with a second stretch regimen that was identical to day 4. This procedure was termed “repetition of a given stretch regimen” and these sheets and their respective unstretched controls were labeled “2×”. After cyclic stretch, the sheets were cut in two equally sized halves. One half was processed for qRT-PCR and one was prepared for fluorescence microscopy.

### MSC seeding and stretching on silicone sheets

Non-reinforced vulcanized matt silicone sheets were obtained from Specialty Manufacturing (No. 70P001-200-030, 40durometer-Shore-A; Saginaw, Michigan, USA). Sheets were coated with fibronectin from bovine plasma (F1141; Sigma-Aldrich, Seinheim, Germany) diluted in PBS (12.5 μg/ml). Silicone sheets (2 × 1 cm) were covered with 700 μl coating substrate in 12-well culture plates for 24 h (room temperature), washed with 0.05% Tween 20 in 2 ml phosphate-buffered saline (PBS), and washed three times with 2 ml PBS. Silicone was seeded with MSCs at passages 3–5 with 15,000 cells/cm^2^, was cultured for 3 days and inserted on day 4 into the bioreactor chamber of the same incubator-housed ElectroForce 5210 BioDynamic-Test-System (Bose, Minnesota, USA). Uniaxial displacement-controlled cyclic stretch was applied at 1 Hz with 10% strain for 16 hours, the sheet was allowed to recover for 8 hours and then stretched again for 16 hours before analysis. After the second cycle of stretch, the sheet was prepared for fluorescence microscopy.

### MSC seeding and stretching in collagen type I hydrogels

Solutions of human MSC (with a final concentration of 1.0 million cells/mL) combined with 5× concentrated DMEM, 10% fetal bovine serum, and acid-solubilized Type I bovine collagen (MP Biomedicals, Solon, OH) was brought to neutral pH with 0.1 M NaOH. The final solution was pipetted into a bioreactor chamber designed and built by Tissue Growth Technologies (TGT LigaGenTM, Minnetonka, MN). The chambers were incubated at 37 °C to permit gelation and an hour later 1× DMEM supplemented with 10% FBS (Lonza) and 1% Penstrep (Lonza) was added. The chambers were cultured for 3 days before the casting mold was removed, leaving the gel suspended. The next day the chamber was inserted in a bioreactor also designed by Tissue Growth Technologies and proprietary software was used to stretch the gel at 10% strain over night before being relieved of stretch and then then stretched for a consecutive night. After the second cycle of stretch, the gel was prepared for fluorescence microscopy.

### Fluorescence microscopy for cell shape

The MSC-seeded sheets used for imaging were stained with Calcein and Hoescht Solution (Cell Viability Imaging Kit, Roche) based on the manufacturer’s protocols and on our previous work^21^. MSCs attached to the collagen sheets were imaged using a Zeiss LSM510 microscope with AxioVison4.8 and manual exposure correction. Calcein-stained MSC morphology was visualized using the green filter and the nuclei were imaged using a blue filter. Using the MosaiX module, a mosaic of 10 × 10 images (with a size of 12,633 × 9,429px corresponding to 8,211.45 × 6,128.85 µm using a 10× objective) were stitched together for analysis. Using the stitched mosaic images, the individual shapes of large quantities of MSCs were counted and measured simultaneously with an ImageJ macro, according to ref. 21. The following four mathematical shape descriptors were quantified for each MSC: length (major axis), circularity (4 * π(area/perimeter^2^), roundness (4 * area/(π * major axis length^2^)), and cell angle (major axis angle relative to the direction of stretch/horizontal axis for controls). These measures were used to quantitatively compare the morphology of MSC populations stretched with different regimens and examples are given in Fig. 1.

### Fluorescence microscopy for protein based analysis

Samples were fixed with 4% formaldehyde at 4 °C for 10 min, washed three times with PBS, and permeabilized with Triton X100 for 20 min, washed three times with PBS, incubated for 1 h with 1:500 rabbit monoclonal anti-calponin (ab46794, Abcam) diluted in PBS containing 0.1% BSA (Sigma-Aldrich), as described in ref. 24. After overnight incubation at 4 °C, the samples were washed 3× with PBS, and incubated with secondary antibody (Goat anti-Rabbit IgG, sc-3739, Santa Cruz Biotechnology, dilution: 1:100) for 1 h at room temperature. Cells washed 3× with PBS, mounted with mounting medium (Dako Denmark A/S), and digitally recorded in a top-down view (Zeiss LSM 510, AxioVison 4.8, manual exposure correction). Thresholds were used to identify MSCs with pixel intensities above 80 (out of 255). These were termed MSCs with a “strong” calponin signal, whereas MSCs with pixel intensities below 80 were termed “weak”. The MSC locations of strong and weak MSC signals were recorded to allow comparing their calculated shape descriptors (see above).

### Quantitative RT-PCR

Compressed collagen sheets with cultured MSCs were digested for 4 minutes at 55 °C using Proteinase K (Fermentas/ThermoScientific). mRNA was isolated using the RNA-Extraction-RNeasy-Minikit (Qiagen, USA). cDNA was synthesized using the Advantage RT-for-PCR Kit (Clontech, USA). Quantitative RT-PCR was performed with the LightCycler 480 SybrGreen Master (catalog no. 04707516001, Roche) and LightCycler 480 Probes Master (no. 04707494001, Roche) using the LightCycler 480 system and Multiwell 96Plates (no. 04729692001, Roche), as described in^21, 24, 53^. Gene expression levels of alpha smooth muscle actin (ACTA2), transgelin (TAGLN), calponin (CNN1), peptidylproplyl isomerase A (PPIA), and human glycerinaldehyde-3-phosphate-dehydrogenase (GAPDH) were determined according to MIQE guidelines^54^. PPIA and GAPDH were used as reference genes. As positive controls and calibrator samples human bladder derived smooth muscle cells (HBdSMC, Promocell, Heidelberg, Germany) were used, as described in ref. 24. The oligonucleotide primers used in qRT-PCR assays were TTGCCTGATGGGCAAGTGAT (forward primer sequence) and TACATAGTGGTGCCCCCTGA (reverse primer sequence) for ACTA2, AGATGGCATCATTCTTTGCGA and GCTGGTGCCAATTTTGGGTT for CNN1, CTCTGCTCCTCCTGTTCG and ACGACCAAATCCGTTGACTC for GAPDH, and TTCATCTGCACTGCCAAGAC and TCGAGTTGTCCACAGTCAGC for PPIA. For TAGLN, the Quiagen assay Hs_TAGLN_2_SG (QT01678516) was used. For TAGLN and PPIA, SybrGreen (Roche) was used. For ACTA2, CNN1, and GAPDH the Roche Universal Probe Library Probes N58 (ACTA2, catalog no. 04688554001), N71 (CNN1, no. 04688945001), and N60 (GAPDH, no. 04688589001) were used, as described in refs 21 and 24.

### Western blotting

MSCs were harvested and cells were lysed in 200 µl protein lysis buffer (40 mM Tris/HCl pH 7.4, 300 mM NaCl, 2 mM EDTA, 2% Triton-X-100) supplemented with proteinase inhibitor at 4 °C. Insoluble material was removed by centrifugation. The protein concentration in the supernatant was determined by Bradford protein assay. Protein samples (50 μg) were separated by 10% SDS-PAGE and transferred to a hydrophobic polyvinylidene difluoride (PVDF) membrane (Immobilon-P; Merck KGaA, Darmstadt, Germany). After blocking with 5% powdered milk (Carl Roth, Karlsruhe, Germany) in TBS-T, membranes were incubated with primary antibodies (rabbit anti-α-smooth muscle actin (SMA), 1:1000 (Abcam), rabbit anti-calponin (CNN),1:1000 (Abcam), rabbit anti-SM22 alpha (SM22), 1:1500 (Santa Cruz), and rabbit anti-β-tubulin, 1:2000 (Cell Signaling, Danvers, MA, USA)) with gentle shaking overnight at 4 °C according to the manufacturer’s protocols. Membranes were washed three times with TBS-T. Secondary antibody (horseradish peroxidase-conjugated anti-rabbit pAb, 1:20,000, Jackson Immuno Research) was added for 2 h at room temperature, and the membranes were washed another three times with TBS-T. Proteins were detected using ECL Western blotting substrate (Thermo Scientific) with membranes exposed to Amersham Hyperfilm ECL (GE Healthcare, Pittsburgh, PA, USA). A pre-stained protein ladder (PageRuler Plus; Thermo Scientific) was used for determination of molecular weights. For quantification, ImageJ (NIH) was used.

### Statistical Analyses

Data are presented as mean ± standard error. All data was graphed and statistically analyzed in SigmaPlot (Systat, Chicago). Data were analyzed for normality. Normally distributed data were compared with a One Way ANOVA test otherwise the data were analyzed with an ANOVA on Ranks test. If the ANOVA revealed significant differences, Dunn’s Method was used for post-hoc analyses to compare individual groups to one another. Statistical differences were indicated with a bar but only when relevant in the presented context; for instance, when examining the effects of time, cells stretched for one vs. two stretch regimens were compared and resulting significant differences related to time were indicated with a bar. In this example, significant differences between different stretch amplitudes (5% vs. 10%) were not indicated, as the analysis focused on the effect of time, not amplitude.
